# A Theoretical Note on the Vital and Toxic Theories of Cancer, and Their Bearing on the Parasitic Theory1Abstracted by the author from the report of the chemical department of the New York State Cancer Laboratory, presented to the Legislature, January, 1903.

**Published:** 1903-08

**Authors:** G. H. A. Clowes

**Affiliations:** Buffalo, N. Y.; Of the Gratwick Research Laboratory University of Buffalo


### A Theoretical Note on the Vital and Toxic Theories of Cancer, and their Bearing on the Parasitic Theory.[1]

By G. H. A. CLOWES, Ph. D., Buffalo, N. Y.
Of the Gratwick Research Laboratory University of Buffalo.

THE theory recently has been advanced in several prominent publications, that the proliferation of the cells of malignant tumors may be attributed to the action either of toxins, or of chemical bodies resulting from abnormal cell metabolism. This statement usually is made without further qualification, leaving the reader to form his own opinion regarding the exact nature of the reaction involved. It must, however, be borne in mind that a toxin or chemical body capable of producing the results observed in cancer could scarcely be the product of the normal or abnormal metabolism of human cells, without the intervention of some external agency; and it is with the intention of showing that the phenomena in question most readily may be attributed to the intervention of some form of parasite, that a few points worthy of interest will be briefly considered.

If we are willing to assume, and it scarcely can be doubted in view of the existing evidence, that some toxic or chemical

1. Abstracted by the author from the report of the chemical department of the New York State Cancer Laboratory, presented to the Legislature, January, 1903.

body plays an important role in causing the cells of malignant tumors to proliferate, the question may well be asked, from what source is this body derived and how does it exert its specific action on the cell? Three possible alternatives may be considered.

1. Some interference with its normal function gives rise to the production within the cell of a toxin or chemical body, capable of exerting a specific action upon the cell, and thereby stimulating it to abnormal development.

2. Some toxin generated possibly at a considerable distance from the point at which the tumor commences, but possessed of an affinity for that particular type of cell, is absorbed by the latter and stimulates it to proliferate.

3. A parasite infecting the cell and itself proliferating and producing a toxin capable of exerting an irritating action on the host cell, causes the latter to proliferate.

The first and second alternatives may readily be disposed of in view of the following facts: a toxin can never, so far as we know, reproduce itself or aid in its own reproduction. It either produces no results, or it exerts a more or less pronounced action upon the cell exposed to it; if that action is too great for the cell to overcome, the latter succumbs; if, on the contrary, the cell is capable of counteracting its toxic activity, an antitoxin probably will be produced. A similar line of reasoning applies to the action of a simple chemical body which is capable of exerting a poisonous action. It reacts with those groups in the cell protoplasm for which it possesses the maximum affinity, and if the quantity introduced is sufficient the cell is destroyed. If this is not the case, the chemical body in question, together with those portions of the protoplasm which have been disturbed by its action, is removed in the ordinary course of cellular metabolism; and unless a fresh supply of the same body is introduced in continually increasing quantities the stimulation of the cell by this means ceases.

In view of these facts it is evident that even were it possible for a toxin or chemical body, produced under abnormal conditions within the cell itself, to stimulate the latter sufficiently to bring about proliferation, which is highly improbable, the body in question would rapidly become exhausted, or at least diluted to such an extent as to render further proliferation under its influence out of the question. As was stated above, such bodies are quite incapable of helping to reproduce themselves; the reverse rather would be expected. One is therefore, in order to uphold the possibility of the first alternative mentioned above, forced to the conclusion that the fresh cells derived from the original cell which first proliferated, have inherited the power of reproducing within themselves a stimulating agent, toxic or otherwise, under whose influence they themselves may subsequently be forced to undergo further proliferation. Such an assumption is quite unwarranted. No analogy for any such action exists in nature, and any such theory may be summarily dismissed, in view of the fact that the principal difficulty which it presents, that is, the source of supply of the ever increasing quantities of toxic material required to stimulate the increasing number of tumor cells, may so readily be explained by assuming the presence of a parasite, as will be shown later.

A second alternative, that the toxin in question is produced in an entirely different portion of the body from that in which the particular cells involved are located, may also be disposed of by means of similar arguments. It must be borne in mind that this toxin would have to exert a selective action, having an affinity for one particular type of cells, but why then should it select any one particular cell, or, having selected that cell, why should it further show a preference for the derivatives of that particular cell after repeated proliferation has taken place? And, as stated in the argument brought against the possible production of a toxin or chemical body within the cell itself, it is quite inconceivable that such a body introduced from without should lead to the continuous reproduction of itself within the cell in question, and its derivatives. It is, on the contrary, highly probable that an antitoxin or antibody would be produced, or that the cells would become so accustomed to its action as to be less readily stimulated to proliferation than the surrounding cells of the same species.

From these considerations it will be seen that when the cell undergoes subdivision in order to counteract the influence of any poisonous body to the action of which it may be exposed, it is improbable that the derivatives of that cell should be endowed with the faculty of reproducing that particular type of poison and thus become stimulated to further reproduction, or that they should be more susceptible to that particular poison than the surrounding cells of the same type. The opposite result would be expected in both cases. The fact that Loeb[1] succeeded in inducing artificial parthenogenesis by means of certain salt solutions can scarcely be put forward as an argument in favor of the assumption that the cancer cell might be led to proliferate in a similar manner. In any case the cause of the increase in concentration of certain ions and the osmotic pressure, as well as

1. Loeb. Am. Jour. of Physiology, Vol. IV., 1901. Artificial Parthenogenesis, etc.

the marked selective activity exhibited would still present considerable difficulties.

The third and only explanation of proliferation of the cells of malignant tumors as being due to the action of some toxic body, which seems to be in any way borne out by the facts with which we are acquainted, is that the cell in question has become infected with some type of parasite which itself proliferates and produces toxins which, in their turn, react upon the host cell stimulating the latter to undergo subdivision. It must be borne in mind that the reaction taking place under such circumstances between the host cell and the parasite which has been introduced, is one in which the excreta of the parasite play a very important part. Soluble products of this nature emanating from the parasite may be supposed to exert a prejudicial effect upon the forces normally possessed by the cell, whereby the latter is enabled to destroy and assimilate or eject foreign bodies finding their way into its protoplasm. The parasite is thus indirectly protected, and, being once established, naturally proliferates and its excreta and the toxins which it probably produces may be supposed to so stimulate the host cell that the latter undergoes subdivision, whereby the number of parasites per cell and consequently the concentration of toxins is diminished.

It then becomes a question of the relative speed at which parasite and host cell proliferate and the relative toxic effect which they exert on one another, as to which of the three following alternatives takes place: (1) the host cell by continual proliferation eventually destroys the invader; (2) owing to a uniform and continuous subdivision, both of host cell and parasite, an exact condition of equilibrium is maintained, at any rate, for a time; (3) the parasite succeeds in entirely exhausting the cell, in which case the parasite would in all probability form some type of permanent spore which would no longer produce toxins capable of affecting the surrounding tissue. For such a mode of procedure we have a good analogy in the case of certain forms of paramecien, which, on becoming infected by lower types of parasites, proliferate and in that way are often enabled to recover from the infection. (See Metchnikoff, L'Immunite, page 20.)

Another rather ingenious but extremely far-fetched suggestion, is that made by Foulerton,[1] who attempts to account for the proliferation of epithelial cells by assuming that up to that point the cells in question have been restrained in their natural tendency to develop, by the function of some antibody induced by reactive materials, which are continually passing into the

1. The Practitioner, Vol. LXIX., Nos. 1 and 2, 1902.

system from organs undergoing degeneration. This line of argument is so involved and introduces so many assumptions for which we have no analogy, that it is hardly necessary to consider it in comparison with the much simpler explanation of toxic activity, previously referred to in connection with the parasitic theory. It is, however, the writer's intention to treat this subject more fully at a later period.

In dealing with the problem of abnormal cell metabolism found in cancer, one important point must be borne in mind: the work of numerous investigators who have attempted to discover the cause of cancer has, at least, shown that the question regarding the existence or nonexistence of a parasite as the prime factor in this disease, is one which can never be decided, making use of generally accepted bacteriological principles. In just the same way it is necessary to deal with the physiological chemical phase of this subject, in an entirely different manner to that which has proved itself so satisfactory in those cases in which only bacteria and their products are involved. It is not, however, proposed to go further into this subject in the present paper.

*Summary.*—An attempt has been made to show that the parasitic theory of the origin of cancer is not in any sense incompatible with the various toxic or chemical theories; in fact, that the production of the toxins generally recognised as playing an important part in this disease, may most readily be attributed to the introduction of some form of intracellular parasite which, on infecting the cell, proliferates, produces toxins and then indirectly stimulates the host cell to proliferation. This theory suggests an explanation for:

1. The production in ever increasing quantities of some body capable of stimulating the cancer cell to enormous proliferation.

2. The selective action displayed by this body for one particular cell, and the direct descendents of that cell, to the exclusion of others of the same type.

3. The apparent impossibility of infecting healthy individuals with the blood of cancer patients, but only by means of intact cells.

It may also be said that this line of reasoning is in accord with the findings of those who have investigated tumors with a view to discover some form of parasite. In conclusion it may be said that the publication of this paper is due to a desire on the part of the writer to check, if possible, the tendency of some authors to advance vague chemical or toxic theories in explanation of the origin of cancer, in lieu of something more specific.

